# Supplementary material for: A Comprehensive Functional Analysis of OsPEAMT1 and OsPEAMT2 Genes in Rice (Oryza sativa L. ssp. japonica)
Source: Plants (Basel). 2025 Sep 22;14(18):2935. doi: 10.3390/plants14182935 (PMC12473159; doi:10.3390/plants14182935)
Supplement: Supplementary file 1 [file plants-14-02935-s001.zip › plants-3809458-supplementary.pdf]

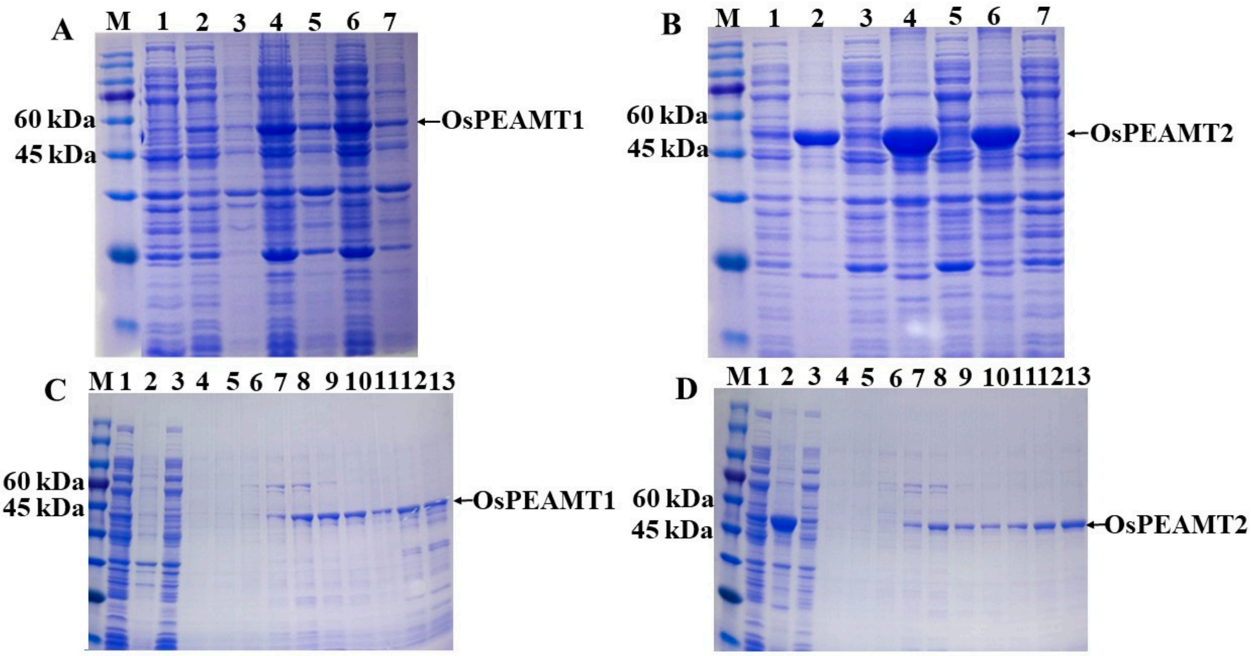


**Figure S1.** Induced expression and purification of OsPEAMT1 and OsPEAMT2 proteins (loading volume of sample was 10 μL). A and B, Isopropyl β-D-1-thiogalactopyranoside (IPTG) induced expression of OsPEAMT1 (A)and OsPEAMT2(B). The pET30a-*OsPEAMT1*-BL21 and pET30a-*OsPEAMT2*-BL21 were transferred to LB liquid medium containing kalamycin(40 μg/ml), respectively. And cultured at 37°C until the OD reached to 0.4-0.6, 0.2 mM IPTG was added and induced expression of OsPEAMT1 and OsPEAMT2 at 18°C, 28°C or 37°C for overnight respectively. Protein stained with Coomassie Brilliant Blue R-250. M: Protein marker (Sigma, Aldrich). In figures A and B, 1: the supernatant of pET30a-*OsPEAMT1*-BL21(A) and pET30a-*OsPEAMT2*-BL21(B) induced by IPTG at 18°C; 2: the precipitate of pET30a-*OsPEAMT1*-BL21 (A) and pET30a-*OsPEAMT2*-BL21(B) induced by IPTG at 18°C; 3: the supernatant of pET30a-*OsPEAMT1*-BL21(A) and pET30a-*OsPEAMT2*-BL21(B) induced by IPTG at 28°C; 4: the precipitate of pET30a-*OsPEAMT1*-BL21 (A) and pET30a-*OsPEAMT2*-BL21(B) induced by IPTG at 28°C; 5: the supernatant of pET30a-*OsPEAMT1*-BL21(A) and pET30a-*OsPEAMT2*-BL21(B) induced by IPTG at 37℃;6: the precipitate of pET30a-*OsPEAMT1*-BL21 (A) and pET30a-*OsPEAMT2*-BL21(B) induced by IPTG at 37°C; 7:before IPTG induction of pET30a-*OsPEAMT1*-BL21(A) and pET30a-*OsPEAMT2*-BL21(B). C and D, the purification of recombinant OsPEAMT1(C) and OsPEAMT1(D), 1: the supernatant after the cells lysis of pET30a-*OsPEAMT1*(C) and pET30a-*OsPEAMT2*(D);2: the precipitate after the cells lysis of pET30a-*OsPEAMT1*(C) and pET30a-*OsPEAMT2*(D); 3: the flow-through fluid of pET30a-*OsPEAMT1*-BL21(C) and pET30a-*OsPEAMT2*-BL21(D); 4-13 represent the elution of OsPEAMT1(C) and OsPEAMT2(D) using 25, 50, 75, 100, 125, 150, 175, 200, 225 and 250 mM of imidazole, respectively.

Supplementary legend for Figure S1

OsPEAMT1, *Oryza sativa* PEAMT1 (NP_001043957.1); OsPEAMT2, *Oryza sativa* PEAMT2 (BAH01482.1); AtPEAMT1, *Arabidopsis thaliana* PEAMT1 (NP_188427.2); AnPEAMT1, *Atriplex nummularia* PEAMT1 (BAD80838.1); BvPEAMT1, *Beta vulgaris* PEAMT1 (BAE07178.1); BnPEAMT1, *Brassica napus* PEAMT1 (AAP83582.1); GhPEAMT1, *Gossypium hirsutum* PEAMT1 HcPEAMT1,*Halostachys caspica* PEAMT1(AHX22003.1); LmPEAMT1, *Leymus mollis* PEAMT1 (AMB37306.1); LbPEAMT1, *Lycium barbarum* PEAMT1 (AGI56231.1); NaPEAMT1, *Nicotiana attenuate* PEAMT1 (OIT39968.1); SePEAMT1, *Salicornia europaea* PEAMT1 (ABG57185.1); SlyPEAMT1, *Solanum lycopersicum* PEAMT1 (AAG59894.1); SoPEAMT1, *Spinacia oleracea* PEAMT1(AAF61950.1); SjPEAMT1, *Suaeda japonica* PEAMT1 (BAC57432.1); SlPEAMT1, *Suaeda liaotungensis* PEAMT1(ABK42071.1); SmPEAMT1, *Suaeda maritima* PEAMT1

(AFW04224.1); TaPEAMT1, *Triticum aestivum* PEAMT1 (AAL40895.1); ZmPEAMT1, *Zea mays* PEAMT1 (AAV67950.1).

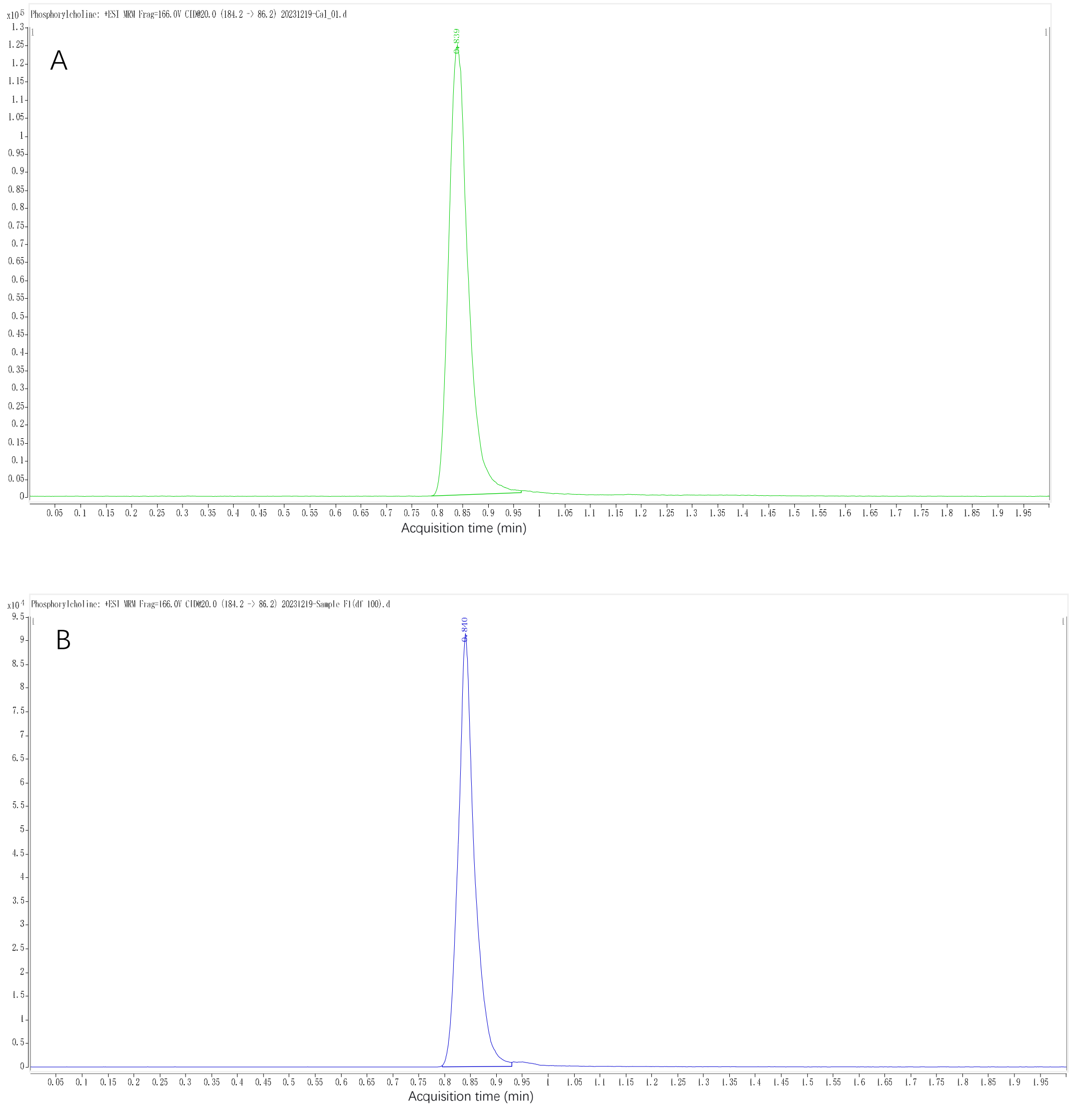


**Figure S2.** Standard solution (A) and sample extracted ion chromatogram (B)

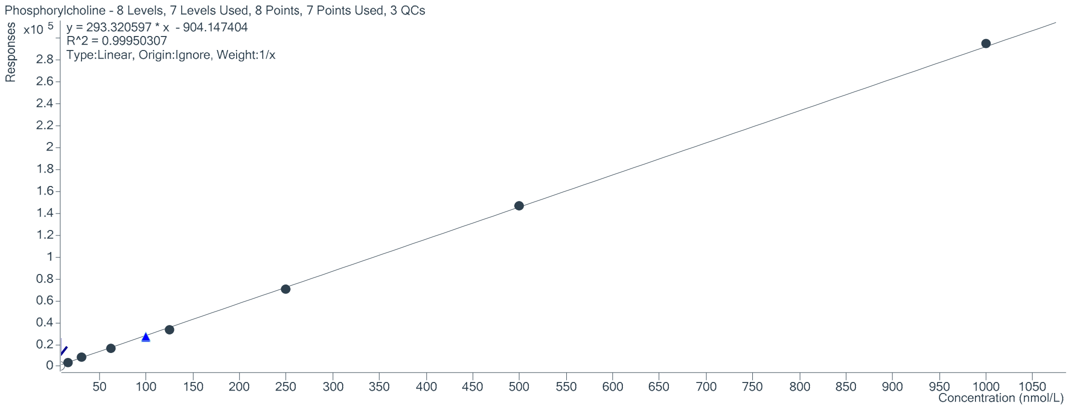


**Figure S3.** The calibration curve of the reference standard for phosphocholine determination by LC-MS/MS.

Supplementary legend for Figure S3

STRE:stress responsive element; G-box: *cis*-acting regulatory element involved in light responsiveness; CGCTA-motif: *cis*-acting regulatory element involved in the MeJA-responsiveness; dOCT: development related element; TCA-element: *cis*-acting element involved in salicylic acid responsiveness; Box4: part of a conserved DNA module involved in light responsiveness; TATC-box: Gibberellin responsive element; LTR: cis-acting element involved in low-temperature responsiveness; ACE: cis-acting element involved in light responsiveness;TGACG-motif:cis-acting regulatory element involved in the MeJA-esponsiveness; ABRE: cis-acting element involved in the abscisic acid responsiveness;TGA-element:auxin-responsive element; P-box: gibberellin-responsive element; DRE core:dehydration responsive element; MBS: MYB binding site;CCAAT-box:MYBHv1 binding site; CCGTCC motif: cis-acting regulatory element involved in the MeJA-responsiveness; ERE: cis-acting regulatory element involved in ethylene responsiveness; GARE-motif: gibberellin responsive element.

**Table S1.** The elution gradient of UHPLC separation

| Time (min) | Solvent A | Solvent B | Flow(μL/min) |
|---|---|---|---|
| 0.0 | 99% | 1% | 300 |
| 2.0 | 99% | 1% | 300 |
| 5.0 | 5% | 95% | 300 |
| 7.0 | 5% | 95% | 300 |
| 7.1 | 99% | 1% | 300 |
| 10.0 | 99% | 1% | 300 |

**Table S2.** The details of *cis*-acting elements of *OsPEAMT1* promoter

| name | sequence | Number | function |
|---|---|---|---|
| $O_2$-site | GATGATGTGG | 1 | *cis*-acting regulatory element involved in zein metabolism regulation |
| dOCT | CTCGGATC | 1 | development related element |
| ABRE | ACGTG | 3 | *cis*-acting element involved in the abscisic acid responsiveness |
| TGA-element | AACGAC | 1 | auxin-responsive element |
| TCA-element | CCATCTTTTT | 1 | *cis*-acting element involved in salicylic acid responsiveness |
| GARE-motif | TCTGTTG | 2 | gibberellin-responsive element |
| ERE | ATTTTAAA | 2 | *cis*-acting regulatory element involved in ethylene responsiveness |
| P-box | CCTTTTG | 2 | gibberellin-responsive element |
| STRE | AGGGG | 6 | stress responsive element |
| WUN-motif | AAATTACT; AAATTTCTT | 2 | wound-responsive element |
| LTR | CCGAAA | 1 | *cis*-acting element involved in low-temperature responsiveness |
| WRE3 | CCACCT | 1 | wound-responsive element |
| MBS/Myb | CAACTG | 1 | MYB binding site involved in drought-inducibility |
| Box 4 | ATTAAT | 2 | part of a conserved DNA module involved in light responsiveness |
| G-box | CACGTC | 4 | *cis*-acting regulatory element involved in light responsiveness |
| TCT-motif | TCTTAC | 1 | part of a light responsive element |
| GATA-motif | AAGGATAAGG | 1 | part of a light responsive element |
| I-box | GTATAAGGCC | 1 | part of a light responsive element |
| ACE | GACACGTATG | 1 | *cis*-acting element involved in light responsiveness |


| name | sequence | Number | function |
|---|---|---|---|
| $O_2$-site | GATGATGTGG | 1 | *cis*-acting regulatory element involved in zein metabolism regulation |
| dOCT | CTCGGATC | 1 | development related element |
| ABRE | ACGTG | 3 | *cis*-acting element involved in the abscisic acid responsiveness |
| TGA-element | AACGAC | 1 | auxin-responsive element |
| TCA-element | CCATCTTTTT | 1 | *cis*-acting element involved in salicylic acid responsiveness |
| GARE-motif | TCTGTTG | 2 | gibberellin-responsive element |
| ERE | ATTTTAAA | 2 | *cis*-acting regulatory element involved in ethylene responsiveness |
| P-box | CCTTTTG | 2 | gibberellin-responsive element |
| STRE | AGGGG | 6 | stress responsive element |
| WUN-motif | AAATTACT; AAATTTCTT | 2 | wound-responsive element |
| LTR | CCGAAA | 1 | *cis*-acting element involved in low-temperature responsiveness |
| WRE3 | CCACCT | 1 | wound-responsive element |
| MBS/Myb | CAACTG | 1 | MYB binding site involved in drought-inducibility |
| Box 4 | ATTAAT | 2 | part of a conserved DNA module involved in light responsiveness |
| G-box | CACGTC | 4 | *cis*-acting regulatory element involved in light responsiveness |
| TCT-motif | TCTTAC | 1 | part of a light responsive element |
| GATA-motif | AAGGATAAGG | 1 | part of a light responsive element |
| I-box | GTATAAGGCC | 1 | part of a light responsive element |
| ACE | GACACGTATG | 1 | *cis*-acting element involved in light responsiveness |

**Table S3.** The details of *cis*-acting elements of *OsPEAMT2* promoter

| name | position | Number | function |
|---|---|---|---|
| AAGAA-motif | GAAAGAA | 1 | involved in secondary xylem development |
| as-1 | TGACG | 2 | involved in transcriptional activity of genes |
| CAT-box | GCCACT | 2 | *cis*-acting regulatory element related to meristem expression |
| MSA-like | (T/C)C(T/C)AACGG(T/C)TA | 1 | *cis*-acting element involved in cell cycle regulation |
| CCAAT-box | CAACGG | 1 | MYBHv1 binding site |
| ABRE | GACACGTGGC; CACGTG | 3 | *cis*-acting element involved in the abscisic acid responsiveness |
| CGTCA-motif | CGTCA | 2 | *cis*-acting regulatory element involved in the MeJA-responsiveness |
| ERE | ATTTCATA | 1 | *cis*-acting regulatory element involved in ethylene responsiveness |
| TGACG-motif | TGACG | 2 | *cis*-acting regulatory element involved in the MeJA-responsiveness |
| TATC-box | TATCCCA | 1 | Gibberellin responsive element |
| P-box | CCTTTTG | 1 | Gibberellin responsive element |
| TCA-element | CCATCTTTTT | 2 | *cis*-acting element involved in salicylic acid responsiveness |
| DRE core | GCCGAC | 1 | dehydration responsive element |
| CCGTCC motif | CCGTCC | 1 | *cis*-acting regulatory element involved in the MeJA-responsiveness |
| W box | TTGACC | 1 | Fungal elicitor responsive element |
| LTR | CCGAAA | 1 | *cis*-acting element involved in low-temperature responsiveness |
| WRE3 | CCACCT | 2 | wound-responsive element |
| MBS | CAACTG | 1 | MYB binding site involved in drought-inducibility |
| box S | AGCCACC | 2 | wound-responsive element |
| Box 4 | ATTAAT | 2 | part of a conserved DNA module involved in light responsiveness |
| G-box | CACGTG | 2 | *cis*-acting regulatory element involved in light responsiveness |
| AE-box | AGAAACAA | 1 | part of a module for light response |
| ACE | CTAACGTATT | 1 | *cis*-acting element involved in light responsiveness |
| ATC-motif | AGTAATCT | 1 | part of a conserved DNA module involved in light responsiveness |
| 3-AF1 | TAAGAGAGGAA | 1 | light responsive element |
| Sp1 | GGGCGG | 1 | light responsive element |

Table S4. The MRM parameters of the target compound

| Analyte | Precursor | Product | CE/V | Polarity |
|---|---|---|---|---|
| Phosphorylcholine | 184.2 | 86.2 | 16 | Positive |
